# Value of additional strain analysis with feature tracking in dobutamine stress cardiovascular magnetic resonance for detecting coronary artery disease

**DOI:** 10.1186/s12968-014-0072-2

**Published:** 2014-10-01

**Authors:** Christopher Schneeweis, Jianxing Qiu, Bernhard Schnackenburg, Alexander Berger, Sebastian Kelle, Eckart Fleck, Rolf Gebker

**Affiliations:** Department of Internal Medicine/Cardiology, German Heart Institute Berlin, Augustenburger Platz 1, 13353 Berlin, Germany; Department of Radiology, Peking University First Hospital, Beijing, China; Philips Research Hamburg, Hamburg, Germany

**Keywords:** Dobutamine stress cardiovascular magnetic resonance, Coronary artery disease, Feature tracking, Circumferential strain, Wall motion abnormality

## Abstract

**Background:**

Dobutamine stress cardiovascular magnetic resonance (DS-CMR) has been established for the detection of coronary artery disease (CAD). The novel technique feature tracking (FT) analyses left ventricular circumferential strain (Ecc) thus offering detailed information about myocardial deformation. The purpose of this study was to evaluate FT based Ecc for the detection of myocardial ischemia during DS-CMR.

**Methods:**

A total of 25 patients (18 males; mean age 64 ± 10 years) with suspected or known CAD underwent a standardized high-dose DS-CMR protocol at 1.5 T. For FT analysis cine short axis (SAX) views (apical, medial, basal) at rest and during maximum dobutamine stress were used. None of the patients had wall motion abnormalities (WMAs) or impaired left ventricular function at rest or scar tissue. For analysis of Ecc the three SAX planes were divided into 16 segments (n = 400 segments). During stress 15 patients (34 segments) developed WMAs as assessed by visual analysis. All patients underwent x-ray coronary angiography for clinical reasons which served as the reference standard. Patients without WMAs during DS-CMR and exclusion of stenotic CAD were defined as normal (10 patients, 160 segments). In patients with significant CAD segments that were supplied by a vessel of >70% narrowing were defined as stenotic (n = 64). The remaining segments in patients with significant CAD were considered as remote (n = 176).

**Results:**

At rest no differences in Ecc were observed between normal, stenotic and remote segments. High-dose dobutamine stress revealed highly significant differences between Ecc of normal and stenotic segments (p < 0.001), as well as between remote and stenotic segments (p < 0.001). The same observation took place for the absolute change of Ecc (p < 0.001 and p = 0.01). ROC analysis of Ecc during maximum DS-CMR differentiated normal from stenotic segments with a sensitivity of 75% and specificity of 67% using a cutoff -33.2% with an area under the curve of 0.78. Additional analysis of intermediate-dose dobutamine also showed a significant difference between normal and stenotic segments (p = 0.001).

**Conclusion:**

FT based analysis of Ecc during intermediate- and high-dose DS-CMR was feasible and differentiated between stenotic, remote and normal segments. Quantitative assessment of Ecc with FT may improve the diagnostic accuracy of DS-CMR for detection of ischemia.

## Background

High-dose dobutamine stress cardiovascular magnetic resonance (DS-CMR) has been established as a method with high diagnostic accuracy for the evaluation of patients with suspected myocardial ischemia [1,2]. Steady state free precession (SSFP) cine imaging during DS-CMR provides a high contrast between intracavitary blood and the endocardium, resulting in a consistently high capability to evaluate myocardial thickening and detection of wall motion abnormalities (WMAs). The assessment of WMAs tends to be subjective as it is based on visual analysis and the clinical experience of the observer. Futhermore, WMAs appear relatively late during the ischemic cascade. Therefore, newer techniques which provide quantitative information about myocardial deformation may be helpful to detect deteriorating myocardial motion at an earlier point in time. Myocardial tagging has shown promising results to improve the detection of ischemia [3], but has been limited by acquiring additional sequences and time consuming post processing. The novel technique feature tracking (FT) analyses left ventricular myocardial strain [4] on the basis of conventional cine images. It was shown that FT is able to detect quantitative changes of wall motion at rest and during low dose dobutamine in healthy volunteers [5] as well as in patients with ischemic cardiomyopathy [6]. However, data on the utility of myocardial strain during high-dose DS-CMR are limited to a few tagging studies [7] and most of them are based on visual or semi-quantitative assessment only [8]. Currently no information exists about the capability of FT for the detection of ischemia during high-dose DS-CMR. Hence, the purpose of this study was to assess FT based left ventricular circumferential strain (Ecc) during DS-CMR and to test its ability to differentiate between stenotic, remote and normal segments.

## Methods

### Patient population

In this retrospective study we included DS-CMR examinations of 25 patients (18 males; mean age 64 ± 10 years) examined for suspicion or progression of known CAD (1 patient). The study was conducted in accordance with the ethical standards defined by local law. In general, written informed consent from patients was obtained before their inclusion in the study. In addition, all participants gave a signature, that their data could be used in anonymized form for scientific work. Further information on patients is given in Table 1. Patients with impaired left ventricular ejection fraction (LVEF), a history of heart surgery, WMA at rest, atrial fibrillation, premature beats, glomerular filtration rate <30 ml/min and/or myocardial scar on Late Gadolinium Enhancement (LGE) were excluded, as well as patients who met contraindications for cardiovascular magnetic resonance (CMR) (e.g. pacemaker, ICD, claustrophobia). Examinations with severe artefacts and low image quality were not considered for this study.

**Table 1:** **Shows demographic details of patients, hemodynamic data and main CMR measurements**

	**Positive DSMR (n = 15)**	**Negative DSMR (n = 10)**
Age (years)	66 ± 8	59 ± 12
Female	3	4
Hypertension	15	7
Diabetes mellitus	3	5
Hyperlipedemia	12	4
Known CAD	1	0
Time between DSMR and coronary angiogramm (weeks)	4 ± 12	8 ± 20
*Hemodynamic data*		
Peak heart rate (bpm)	136 ± 12	143 ± 9
Heart rate at rest (bpm)	66 ± 9	70 ± 9
Peak BP (mmHg)	146/73 ± 28/13	131/68 ± 17/9
BP at rest (mmHg)	141/76 ± 22/14	129/71 ± 15/13
Maximum dobutamine dose (μg/kg/min)	36 ± 7	37 ± 5
Atropin dose (ml)	0.6 ± 0.3	0.25 ± 0.04
*MRI parameter*		
LVEF (%)	61 + 4	62 ± 4
LVEDV (ml)	154 ± 31	143 ± 32
LVESV (ml)	60 ± 14	54 ± 14

### Coronary angiography

Coronary angiography served as reference standard to define stenotic CAD. All patients underwent coronary angiography within 8 ± 20 weeks (no WMA during DS-CMR) and 4 ± 12 weeks (WMA during DS-CMR) after DS-CMR. Patients without WMAs underwent coronary angiography due to clinical indications. Angiograms were evaluated visually for stenosis of all major epicardial coronary arteries and their branches by a highly experienced interventional cardiologist. The severity of coronary stenosis was derived from one single view showing the maximal reduction in absolute luminal diameter. A significant coronary stenosis was defined as ≥70% luminal diameter reduction in vessels with ≥2 mm diameter. In addition, myocardial segments were assigned to the supplying coronary artery based on a consensus read of the interventionalist and the CMR reader taking the respective coronary dominance type into account. Patients without WMAs during DS-CMR and exclusion of CAD by coronary angiography were defined as normal and served as control. The remaining segments in patients with significant CAD were considered as remote.

### CMR image acquisition and analysis

CMR was performed using a conventional 1.5 T magnetic resonance system (Achieva, Philips, Best, The Netherlands) and a 5 array cardiac surface coil. During image acquisition ECG and breathing motion were detected continuously. Cine imaging was performed using balanced SSFP and retrospective gating (repetition time, 3.4 ms; echo time, 1.7 ms; flip angle, 60, in-plane spatial resolution was 1.8 × 1.8 mm with a slice thickness of 8 mm). Patients were instructed to stop beta-blocker medication at least 24 hours before DS-CMR.

All patients underwent a standardized DS-CMR described elsewhere [9]. Standard LGE imaging was performed 10 minutes after the termination of dobutamine infusion. For LGE an intravenous bolus of 0.2 mmol/kg gadolinium-diethylenetriamine-pentaacetic acid (Magnevist, Bayer, Berlin, Germany) was administered.

For CMR image analyses of left ventricular ejection fraction (LVEF), left ventricular end-diastolic volume (LVEDV), and left ventricular end-systolic volume (LVESV) the custom software package (Philips, MR WorkSpace 2.6.3.3) was used. Volumetric analysis of the LV was performed using Simpson’s method. For WMA analysis 2 observers performed a consensus segmental analysis. Both were blinded to the patients’ identities and results of the coronary angiography.

### Feature tracking

Cine balanced SSFP (bSSFP) SAX images (basal, medial and apical) were used for the Ecc analysis with FT. The bSSFP cine images were stored as Digital Imaging and Communication in Medicine (DICOM) and analysed using the CMR FT software (TomTec Imaging Systems, Munich, Germany). Endocardial contours were drawn manually at end-diastole. The software tracks different features and generates information about myocardial deformation throughout the cardiac cycle [4]. In case of unsatisfactory detection of the endocardial border the contour was modified until a satisfying tracing resulted.

One operator, who was blinded to the results of the DS-CMR and coronary angiogram, drew the contours at rest, low- (10 μg), intermediate (20 μg)- and high-dose (40 μg) dobutamine. Additional to comparing segments defined by coronary angiography into normal, stenotic and remote we specifically analyzed those that developed a WMA during DS-CMR. Time to peak Ecc was compared between segments with WMA and remote segments. In order to assess interobserver variability a random subset of rest and stress images were re-analysed in 9 subjects (144 segments) by a second operator.

### Statistical analysis

Statistical analysis was performed using SPSS 18.0 for Windows (SPSS Inc.) and MedCalc. All parameters were given as mean ± standard deviation (SD). Segmental based data were compared using a one-way ANOVA followed by the Scheffé’s post-hoc analysis or in case of not normal distribution with Tamhane’s post-hoc analysis. T-tests were performed for analysis of low-dose and intermediate dose Ecc values. For testing differences of time to peak values the paired t-test was used. For assessment of the interobserver variability the intraclass correlation coefficient and Bland-Altman analysis was used.

## Results

### DS-CMR

In all patients the target heart rate was achieved. All patients tolerated the protocol and no premature termination was necessary. Overall no severe adverse events were observed. Fifteen patients developed a WMA (34 segments) during DS-CMR, in 10 patients no WMA (160 segments) was detectable. All patients showed a normal LVEF. Remaining CMR results and hemodynamic data are reported in Table 1.

### Coronary angiography

**Figure 1:**
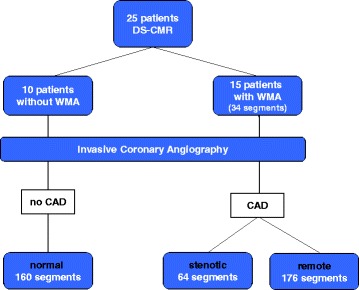
**Flowchart of segmental classification based on the results of DS-CMR and corresponding coronary angiogram.**

All patients underwent coronary angiography. Significant CAD was diagnosed in 15 patients. The remaining 10 patients had no significant CAD and were defined as normal. Based on coronary angiogram 64 segments were defined as stenotic (≥70% stenosis), 176 as remote (no significant stenosis in patients with CAD) and 160 as normal (Figure 1).

### Feature tracking

**Figure 2:**
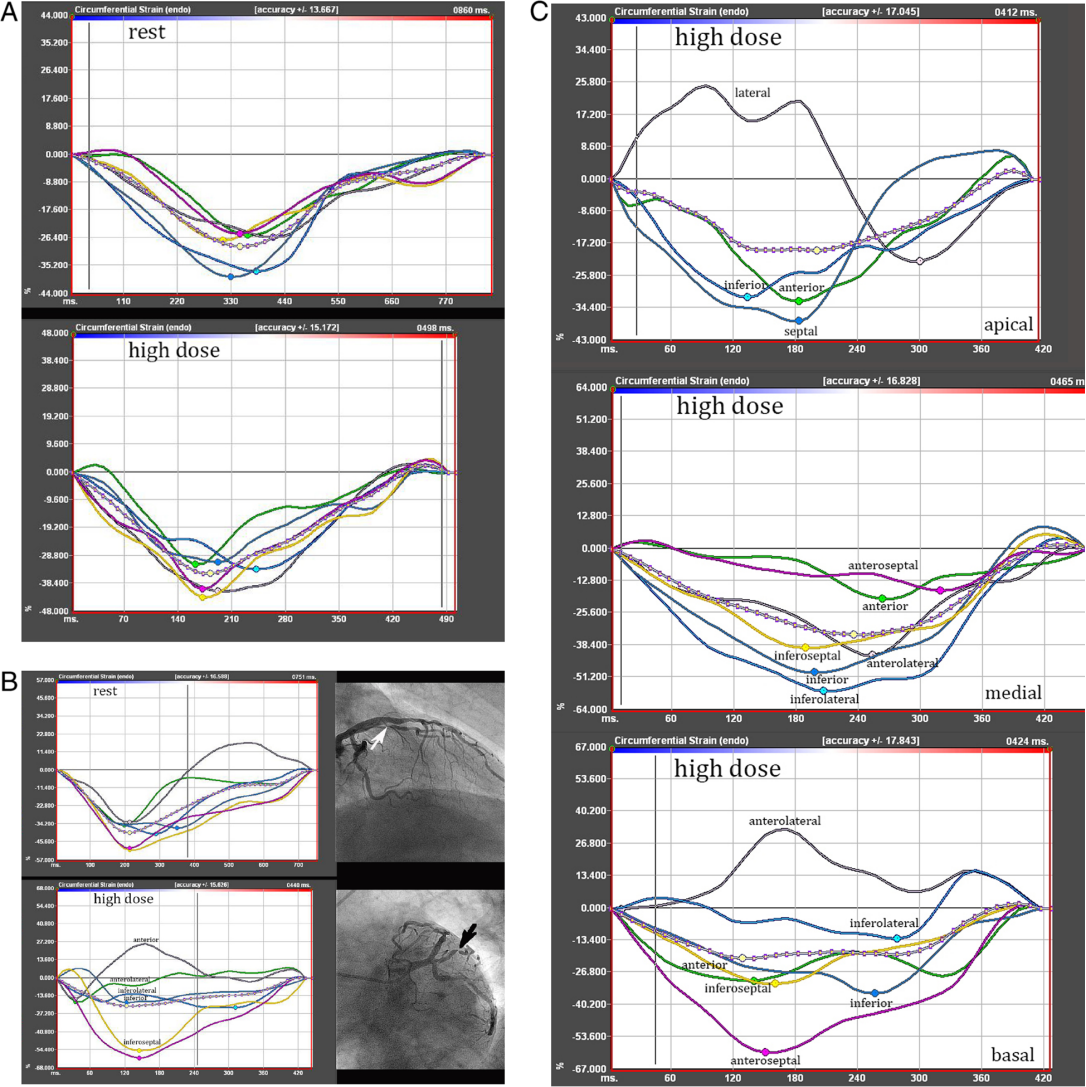
**Segmental strain analysis. A:** Segmental based circumferential strain analysis at rest and during high-dose dobutamine in a patient without WMA and normal coronary angiogram. **B:** Segmental strain analysis in a patient, who developed a WMA of the anterolateral segment. The Ecc of the neighbouring anterior and inferolateral segments was also impaired. The coronary angiogram showed a corresponding high-grade stenosis of the first diagonal branch. **C:** Ecc analysis of basal, medial and apical SAX under high-dose dobutamine stress. The depicted strain curves originate from three different patients, who all developed a WMA. Angiography showed high grade stenosis of the left circumflex artery in the patient at the bottom (basal SAX). Both patients in the middle and at the top had stenoses of the left anterior descending artery.

At rest no differences in Ecc were observed between the different groups (normal: −28.1 ± 8.9%; stenotic: −25.9 ± 10.7%; remote: −27.5 ± 11.6% and WMA: −24.8 ± 12.9%; p = 0.62 for normal vs. stenotic, p = 0.66 for nomal vs. WMAs, p = 0.97 for normal vs. remote and p = 0.79 for remote vs. stenotic segments). Ecc analysis at high-dose dobutamine stress showed highly significant differences between normal and stenotic segments (normal: −39.3 ± 11.3%; stenotic: −23.8 ± 15.9%, p < 0.001, representative imaging examples are given in Figures 2A, 2B and 2C) as well as between normal segments and segments which developed WMAs (−19.2 ± 19.1%, p < 0.001). Furthermore, Ecc differed significantly between normal and remote segments (remote: −33.9 ± 15.1%, p = 0.002), while no difference was observed between segments with WMAs and stenotic segments (p = 0.79). The difference between remote segments and stenotic segments was highly significant (p < 0.001).

Similar results were seen when comparing the absolute change (Δ) of Ecc between rest and stress for normal vs. stenotic segments (Δ normal: −11.2 ± 12.6%, Δ stenotic: 2.1 ± 18.4%; p < 0.001) and vs. segments with WMAs (Δ WMA: 5.6 ± 22.9%; p = 0.001), as well as between normal and remote segments (Δ remote: −6.5 ± 17.2%; p = 0.023). Furthermore, the difference between remote and stenotic segments was significant (p = 0.01) and no difference was detected between segments with WMAs and stenotic segments (p = 0.97).

**Figure 3:**
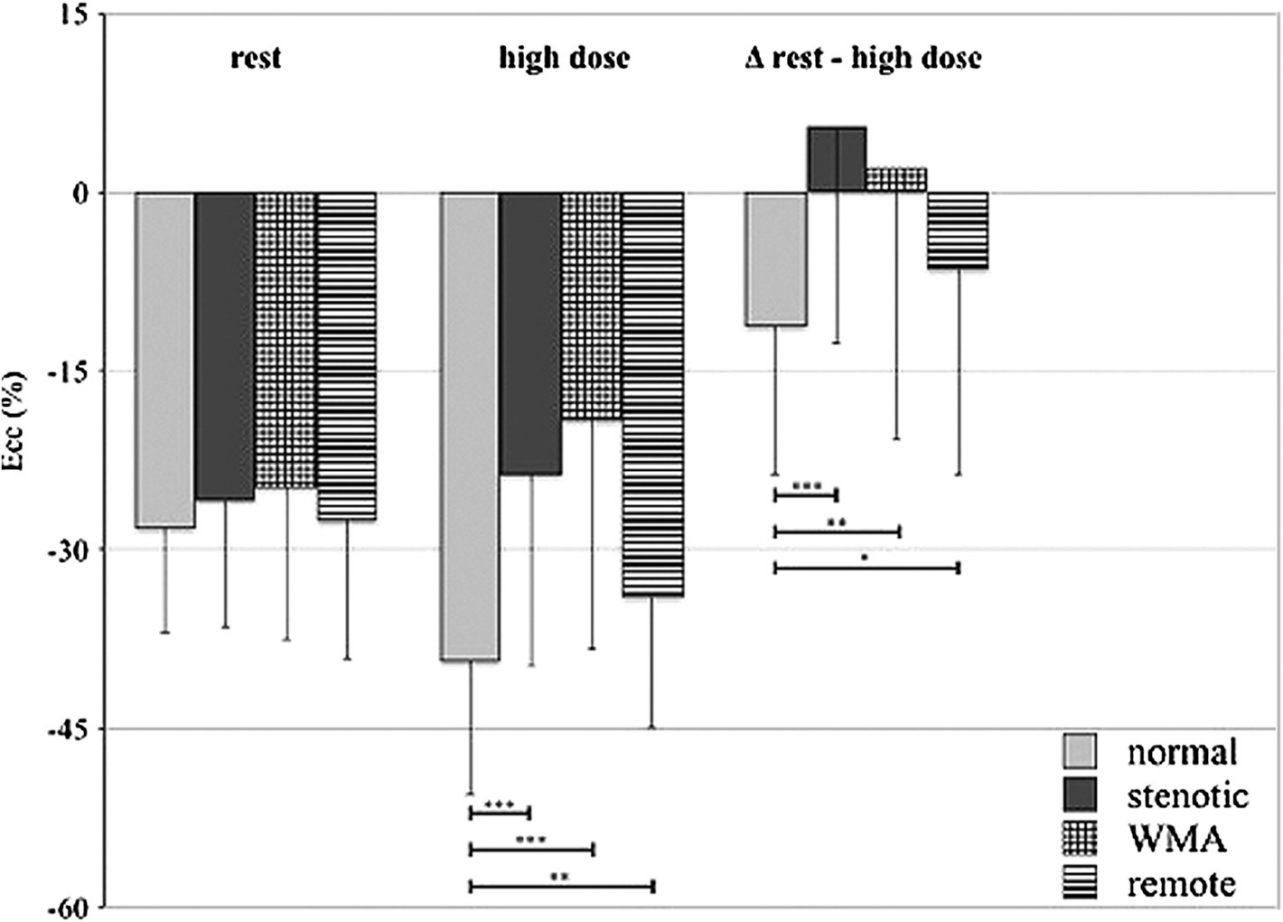
**Bar graphs displaying the circumferential strain values at rest and high-dose dobutamine stress as well as the absolute change (Δ) of Ecc.** Significant differences were observed between normal segments and the remaining segments.

**Figure 4:**
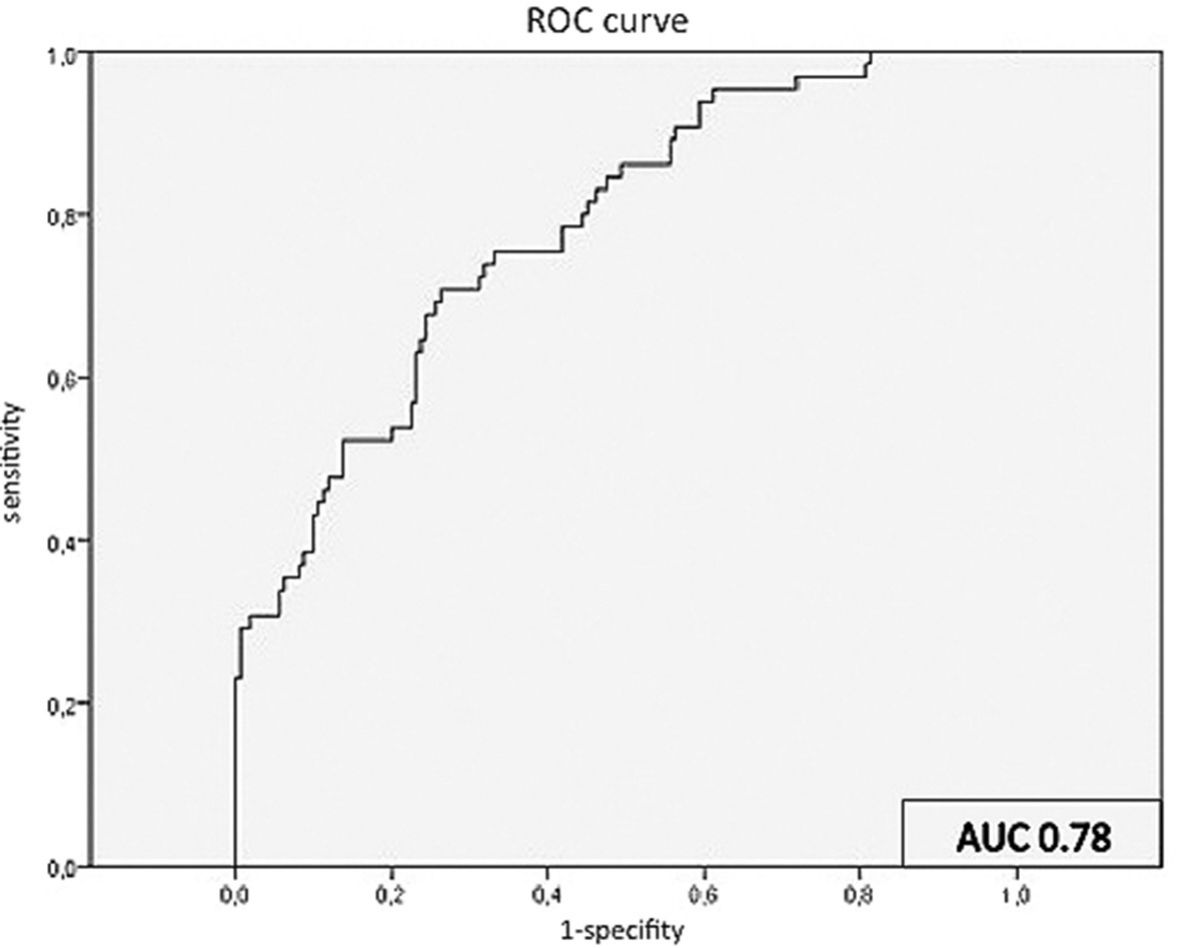
**ROC analysis for Ecc during maximum DS-CMR.** At a cutoff of -33.2% sensitivity and specificity to differentiate between normal and stenotic segments was 75% and 67%, respectively.

Results of the Ecc analysis are summarized in Figure 3. ROC analysis for Ecc during high-dose DS-CMR differentiated normal from stenotic segments with a sensitivity of 75% and specificity of 67% using a cut-off of −33.2% with an area under the curve of 0.78 (95% CI 0.72-0.85) (Figure 4). Due to the observed significant differences between normal and stenotic segments, we performed additional analyses for Ecc at low- and intermediate dobutamine stress. At low-dose dobutamine there was no difference between normal and stenotic segments (−32.9 ± 11%; −30.1 ± 9.6%; p = 0.08) However, at intermediate-dose dobutamine stress normal and stenotic segments differed significantly (−39.5 ± 10.4%; −33.9 ± 13.2%; p = 0.001). In regard to the absolute change (Δ normal: 11.4 ± 11.5%; Δ stenotic: 8.5 ± 14.9%) the difference was not statistically significant (p = 0.18).

In segments with WMA time to peak Ecc was longer than in remote segments (203.4 ± 56.8 ms vs. 163.7 ± 41.4 ms, p = 0.007).

### Interobserver agreement

**Figure 5:**
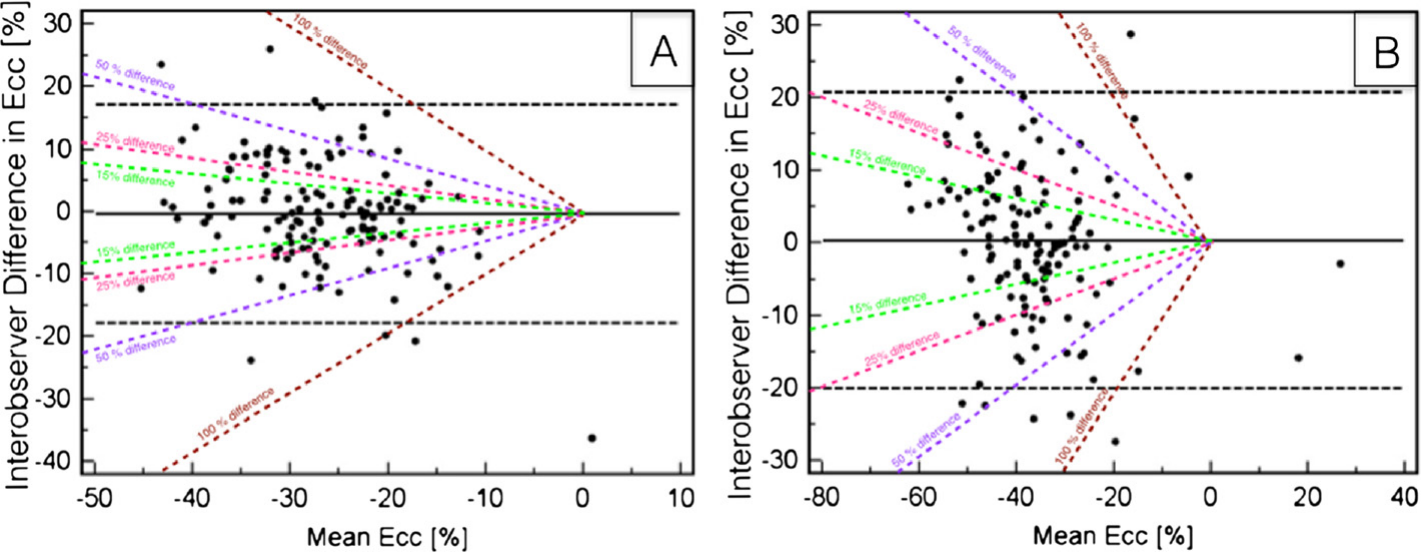
**A and B: Bland Altman plots with bias (solid black line) and limits of agreement (dotted black line) for interobserver agreement at rest (A) and high-dose dobutamine stress (B).** Values are expressed as %. The oblique dashed lines demonstrate 15, 25, 50 and 100% difference levels.

The intraclass correlation coefficients (ICC) indicated a strong agreement for Ecc at rest (0.7) and very high agreement at high-dose dobutamine stress (0.85). The Bland-Altman analyses showed reasonable agreement at rest as well as during high-dose dobutamine stress, but lack of consistent agreement for Ecc values less than −20% at rest and stress. Variability above −20% for rest as well as stress was mainly within 50% difference levels and nearly half of all values were within the 15% difference level (Figure 5A and B).

## Discussion

The aim of this work was to evaluate the ability of FT derived Ecc to detect stress-inducible myocardial ischemia during high-dose DS-CMR. FT is a new method for assessing myocardial strain based on the analysis of cine images. There is growing interest in this method as reflected by a number of recently published studies that compared FT to other strain analysis tools such as myocardial tagging [4] and speckle tracking [10]. Until now the utility of FT for DS-CMR has only been demonstrated using low-dose regimen in healthy volunteers [5] and patients with ischemic cardiomyopathy [6]. The application of FT during low-dose dobutamine or in patients with decreased LV function may be somewhat easier. Due to the increasing myocardial contractility and consecutive partial obliteration of the LV cavity during high-dose dobutamine, discrimination of the endocardial border using FT becomes more challenging. Our study demonstrated that FT was feasible and allowed to distinguish between stenotic, remote and normal segments. The Ecc values from our study at rest were within the range of previously published data [5]. Furthermore, FT demonstrated quantitative changes of Ecc during DS-CMR in segments that developed WMAs based on conventional visual analysis. The significant differences of Ecc in segments with WMA were accompanied by higher time to peak values thus indicating that peak Ecc is not only decreased but also delayed in segments with WMA.

Overall 64 segments were defined as stenotic, while only in 34 segments a WMA was visually detected. Ecc did not show differences between stenotic segments and segments, which developed WMAs. This indicates that Ecc may detect more ischemic segments as compared to the visual analysis of wall motion. Physiologically a perfusion deficit appears as the earliest sign of myocardial ischemia followed by metabolic alterations. With continuation of ischemia these effects become more pronounced and induce diastolic and systolic dysfunction before finally leading to myocardial infarction. Based on this, inducible WMAs during high-dose DS-CMR appear relatively late in the ischemic cascade.

Interestingly, we could show a difference of Ecc between normal and remote segments during high-dose dobutamine stress. Deterioration of strain in stenotic segments may not only be limited to the segment itself and will consequently affect neighbouring segments. Thus, these findings could be an expression of generalized changes of strain in the presence of only a regional coronary narrowing.

Our study demonstrated that the earliest sign of ischaemia detectable by Ecc using FT occurred during intermediate dose dobutamine stress. Differences in Ecc between stenotic and normal segments became even more pronounced at high-dose dobutamine stress. Furthermore, in normal segments strain did not increase further between intermediate and high-dose dobutamine stress. These findings are in line with a previous study that used direct color-coded visualization of myocardial strain with Strain-Encoded MR (SENC) [11]. The implementation of FT in clinical routine may help in earlier detection of ischaemia during intermediate stages, thereby increasing patient safety during pharmacologic stress testing.

Overall the analysis of myocardial strain during high-dose DS-CMR has been limited to a few studies using tagging [3,7,12] and SENC [8]. These studies have shown promising results to improve the accuracy for the detection of myocardial ischemia. However, most data were assessed only visually and quantitative data are only available for the apical SAX [7]. The limited amount of studies using tagging is most likely related to the necessity of acquiring additional sequences and the time-consuming post processing to assess strain values. In contrast, FT can process conventional cine images thus being potentially more time efficient. Previous studies demonstrated that Ecc seems to be the most robust and reproducible strain parameter [13]. For the interobserver agreement we noted a strong agreement for Ecc at rest (0.7) and very high agreement at high-dose dobutamine stress (0.85) when using ICC. Additional analysis with Bland Altman plots showed a reasonable agreement for Ecc values at rest and high-dose and are comparable to published data [5,6]. In our study half of the inter-observer measurements were within the 15% difference level, which implies that FT Ecc analysis on a segmental level becomes challenging at rest, but does not become worse during dobutamine stress.

## Conclusion

The results of FT in DS-CMR seem to be promising and may improve the diagnostic accuracy of DS-CMR for the detection of ischemia. Further studies are necessary to evaluate FT in intermediate and high-dose DS-CMR and to determine whether a robust threshold for discriminating ischemic from non-ischemic segments can be given.

### Limitations

Our study has important limitations. Conventional coronary angiography with visual assessment of severity of stenoses was used rather than fractional flow reserve (FFR) or quantitative coronary angiography (QCA) to distinguish between stenotic, remote and normal segments. The discrepancies between the anatomical and physiological approach are well known which may have introduced a bias to our study. Also, we included patients with relatively good image quality only. Furthermore, patients with previous myocardial infarction were excluded; hence our results may not be applicable to patients with known myocardial infarction and pre-existing WMAs. The results of this study were based on the circumferential strain of the short axis geometry as it seems to be the most reliable and reproducible parameter [14].

## Abbreviations

DS-CMR: Dobutamine stress cardiovascular magnetic resonance
CAD: Coronary artery disease
FT: Feature tracking
Ecc: Circumferential strain
SAX: Short axis
WMA: Wall motion abnormality
SSFP: Steady state free precession
bSSFP: Balanced SSFP
LVEF: Left ventricular ejection fraction
LVEDV: Left ventricular end-diastolic volume
LVESV: Left ventricular end-systolic volume
LGE: Late gadolinium enhancement
CMR: Cardiovascular magnetic resonance
SD: Standard deviation
SENC: Strain-encoded MR
FFR: Fractional flow reserve
